# α-Synuclein A53T Promotes Mitochondrial Proton Gradient Dissipation and Depletion of the Organelle Respiratory Reserve in a Neuroblastoma Cell Line

**DOI:** 10.3390/life12060894

**Published:** 2022-06-15

**Authors:** Pierpaolo Risiglione, Salvatore Antonio Maria Cubisino, Cristiana Lucia Rita Lipari, Vito De Pinto, Angela Messina, Andrea Magrì

**Affiliations:** 1 Department of Biomedical and Biotechnological Sciences, University of Catania, Via S. Sofia 64, 95125 Catania, Italy; pierpaolo.risiglione@phd.unict.it (P.R.); salvatore.cubisino@phd.unict.it (S.A.M.C.); cristiana.lipari@phd.unict.it (C.L.R.L.); vdpbiofa@unict.it (V.D.P.); 2 we.MitoBiotech S.R.L., Corso Italia 172, 95125 Catania, Italy; 3 Department of Biological, Geological and Environmental Sciences, University of Catania, Via S. Sofia 64, 95125 Catania, Italy

**Keywords:** αSyn, mitochondrial dysfunction, Parkinson’s disease, high-resolution respirometry

## Abstract

α-synuclein (αSyn) is a small neuronal protein whose accumulation correlates with Parkinson’s disease. αSyn A53T mutant impairs mitochondrial functions by affecting substrate import within the organelle, activity of complex I and the maximal respiratory capacity. However, the precise mechanism initiating the bioenergetic dysfunction is not clearly understood yet. By overexpressing αSyn A53T in SH-SY5Y cells, we investigated the specific changes in the mitochondrial respiratory profile using High-Resolution Respirometry. We found that αSyn A53T increases dissipative fluxes across the intermembrane mitochondrial space: this does not compromise the oxygen flows devoted to ATP production while it reduces the bioenergetic excess capacity of mitochondria, providing a possible explanation of the increased cell susceptibility observed in the presence of further stress stimuli.

## 1. Introduction

α-synuclein (αSyn) is a small neuronal protein mainly expressed in the brain whose physiological function remains largely unknown. αSyn localizes principally at the synapsis, where it is believed to take part in the release of the neurotransmitter vesicles [1,2]. Despite the lack of an organized structure in solution, evidence suggests that αSyn exists in a dynamic equilibrium between a cytosolic unstructured and an N-terminal α-helix structured form, the last adopted when it binds the membranes [3,4]. A predominantly β-sheet conformation is adopted during αSyn aggregation processes into toxic oligomers and amyloid fibrils [5]. Fibrils are the main component of the Lewy bodies, intraneuronal inclusions typical of a group of neurodegenerative disorders collectively known as synucleinopathies and including Parkinson’s disease (PD), dementia with Lewy bodies and multiple system atrophy [6]. 

Although it is mainly cytosolic, αSyn was detected in several subcellular compartments, such as mitochondria [7]. It has been proposed that αSyn crosses the outer mitochondrial membrane (OMM) by using VDAC pores [8,9]. Within the intermembrane space (IMS), αSyn seems to participate in the modulation of the mitochondrial bioenergetic functions [10]. In mice, the genetic knock-out of the αSyn gene, SNCA, significantly reduces the coupling between the electron transport through the respiratory complexes and the ATP production [11]. However, pathological forms have a harmful impact on mitochondrial functioning. For instance, αSyn aggregates affect ATP synthesis, complex I activity [12,13], and compromise the import of protein and substrates within the organelle [14,15]. 

The transition from a physiological conformation to pathological forms is often associated to missense mutations in SNCA gene. For instance, the substitution of alanine 53 in threonine (A53T) associates to the early onset of familial PD [16]. This mutation increases the rate of αSyn fibrils formation, leaving unvaried the ability of the protein to bind phospholipid membranes [17,18]. As widely reported in the literature, the expression of αSyn A53T correlates with mitochondrial dysfunction. In transgenic mice, A53T mutant accumulation induces mitochondrial DNA damage and the opening of the permeability transition pore [19]. In the same murine model, extensive mitophagy events precede dopamine depletion and neurodegeneration [20]. Furthermore, in primary cortical neurons, A53T promotes a decrease of the maximal respiratory capacity of mitochondria [21]. However, the precise mechanism by which the αSyn mutant initiates the impairment of mitochondrial bioenergetics remains largely unknown. 

With this aim, in this work αSyn A53T was overexpressed in the neuroblastoma SH-SY5Y cell line and the respiratory profile was analyzed. Our findings indicate that overexpression of αSyn A53T, but not of the corresponding wild-type protein, affects the maximal respiratory capacity and promotes an increase of the dissipative-related respiration. This latter does not correlate with a decrease in flows related to mitochondrial ATP production upon stimulation of the oxidative phosphorylation system activity. Importantly, the combination of these events results in a drastic reduction of the energy excess capacity, a respiratory reserve on which mitochondria may draw in presence of stress conditions. 

Overall, our results may help to understand the general impact of αSyn A53T on mitochondrial function and how this mutant may contribute to increase cell vulnerability to additional stress stimuli, such as oxidative stress, typical of PD and other synucleinopathies. 

## 2. Materials and Methods

### 2.1. Cloning and Mutagenesis

The sequence encoding wild-type (WT) human αSyn was amplified by PCR from a stock plasmid and cloned in the modified pCMS-mtDsRED vector (Clontech, Montain View, CA, USA) [22] in frame with the HA-tag at the C-terminal domain. Cloning was performed by PCR using available protocol [23]. The introduction of A53T mutation was achieved by site-directed mutagenesis on pCMS construct carrying αSyn WT by the QuickChange Site-Directed Mutagenesis kit (Stratagene, La Jolla, CA, USA) according to the manufacturer’s instructions. All primers used in amplification, cloning and mutagenesis are listed in Table S1. All sequences were verified by sequencing. 

### 2.2. Cell Line Maintenance, Transfection and Viability

The human neuroblastoma SH-SY5Y cell line was maintained in DMEM medium supplemented with 10% fetal bovine serum, 2 mM L-glutamine and 1% penicillin/streptomycin (Sigma-Aldrich, St. Louis, MO, USA) and kept in a controlled environment (37 °C and 5% CO_2_). Cells were transfected with constructs carrying αSyn sequences or empty vector using K2 Transfection System (Biontex, Munich, Germany) according to manufacturers’ instructions. All experiments were performed after 48 h from transfection. 

Viability of transfected SH-SY5Y cells was determined by MTT assay [24]. Cells were seeded in 96-well plate and the colorimetric reaction was analyzed using the microplate reader Varioskan (Thermo Fisher Scientific, Waltham, MA, USA). For each condition tested, three independent experiments in triplicate were performed. 

### 2.3. Western Blot

Whole lysates from transfected SH-SY5Y were separated on NuPAGE Bis-Tris polyacrylamide gels (Thermo Fisher Scientific, Waltham, MA, USA) and transferred to PVDF membranes (GE Healthcare, Chicago, IL, USA). After blocking, membranes were incubated with the following primary antibodies: anti αSyn (Abcam, Cambridge, UK, cat. no. ab80627, 1:1000), anti VDAC1 (Abcam, Cambridge, UK cat, no. ab14734, 1:1000), anti SDHA (Cell Signaling, Danvers, MA, USA, cat. no. 11998, 1:1000) and anti β-actin (Cell Signaling, Danvers, MA, USA, cat. no. 4970, 1:2000). Afterwards, membranes were incubated with IRDye conjugated secondary antibodies (LI-COR Biosciences, Lincoln, NE, USA, 1:25,000). Signals were detected using Odyssey Imaging System (LI-COR Biosciences, Lincoln, NE, USA). Three independent experiments were performed.

### 2.4. High-Resolution Respirometry (HRR)

Mitochondrial respiration capacity of SH-SY5Y cells was analyzed in intact and permeabilized cells using the two-chamber system O2k-FluoRespirometer (Oroboros Instruments, Innsbruck, Austria) by HRR. All experiments were performed at 37 °C in respirometric buffer Mir05 (Oroboros Instruments, Innsbruck, Austria), under constant stirring. 

The oxygen consumption in the different respiratory states was determined using a specific substrate-uncoupler-inhibitors titration (SUIT) protocol as in [25]. Respiration in intact cells, the ROUTINE state, was first measured. Plasma membranes were permeabilized with the addition of 4 μM digitonin and the LEAK state was measured in the presence of previously added pyruvate (5 mM) and malate (2 mM) but in absence of adenylates. OXPHOS state was achieved by the addition of glutamate and succinate (10 mM) in the presence of saturating concentrations of ADP (2.5 mM). The uncoupled maximal capacity of the electron transport (ET) system was attained by titration with carbonyl cyanide 3-chlorophenylhydrazone (CCCP, 0.5 μM). Afterwards, rotenone (2 μM) was added, allowing the measurement of the complex II activity in the ETS state. The residual oxygen consumption (ROX) was achieved by the addition of antimycin (2.5 μM respectively). All chemicals were purchased from Sigma-Aldrich (St. Louis, MO, USA). Six independent experiments were performed for each condition.

### 2.5. Analysis of Respirometric States

Instrumental and chemical background fluxes were calibrated as a function of oxygen concentration using DatLab software (version 7.4, Oroboros Instruments, Innsbruck, Austria). The rate of oxygen consumption in the respiratory states ROUTINE, LEAK, OXPHOS and ET capacity was corrected for the ROX and expressed as pmol/sec per million cells or as Flux Control Ratios (FCRs) relative to the ET capacity [26]. Oxygen flows relative to net respiration, coupling respiration and excess capacity were calculated as described [25].

### 2.6. Statistical Analysis

Data are expressed as a mean or median ± SD and statistically analyzed by one-way ANOVA following by Tukey’s test using Prism software (version 9, GraphPad Inc., San Diego, CA, USA). Cells transfected with the empty vector were used as control. The values of * *p* < 0.05 and ** *p* < 0.01 were taken as significant.

## 3. Results

### 3.1. Characterization of SH-SY5Y Overexpressing αSyn A53T

**Figure 1:**
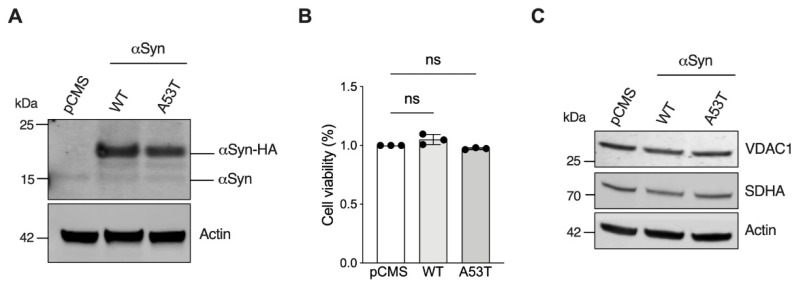
Analysis of αSyn WT and A53T expression in SH-SY5Y cells. (**A**) Representative Western blot of lysates obtained from SH-SY5Y previously transfected with the empty vector (pCMS) or constructs carrying αSyn WT or A53T. Exogenous αSyn expression is shown. Actin was used as loading control. (**B**) MTT assay of transected SH-SY5Y. Data are expressed as a mean ± SD of *n* = 3 independent experiments and analyzed by one-way ANOVA; ns, not significant. (**C**) Representative Western blot of same lysates as in A showing the levels of mitochondrial markers VDAC1 and SDHA. Actin was used as loading control.

In order to investigate the effect of αSyn A53T for mitochondrial respiration, the neuroblastoma cells SH-SY5Y were transiently transfected with a plasmid carrying αSyn A53T or WT encoding sequence, or the empty vector as control (hereafter simply named pCMS). The efficiency of cell transfection was verified after 48 h by Western blot, whereas the overall effect on cell viability was evaluated by MTT assay. As shown in Figure 1A, the transfection efficiently allowed the detection of an intense band at the expected molecular weight, consisting of the exogenous αSyn HA-tagged protein, in addition to the endogenous one. However, in accordance with the previous observation [27], the overexpression of αSyn A53T or WT achieved by transient transfection did not cause a significant loss of cell viability in our cellular model (Figure 1B). 

By assaying the levels of specific mitochondrial markers upon transfection, we next estimated the mitochondrial mass. As displayed in Figure 1C, significant variations among the samples were observed neither for VDAC1 nor for SDHA, proteins located in the OMM and in the inner mitochondrial membrane (IMM), respectively.

### 3.2. αSyn A53T Reduces the Maximal Respiratory Capacity of SH-SY5Y Cells

**Figure 2:**
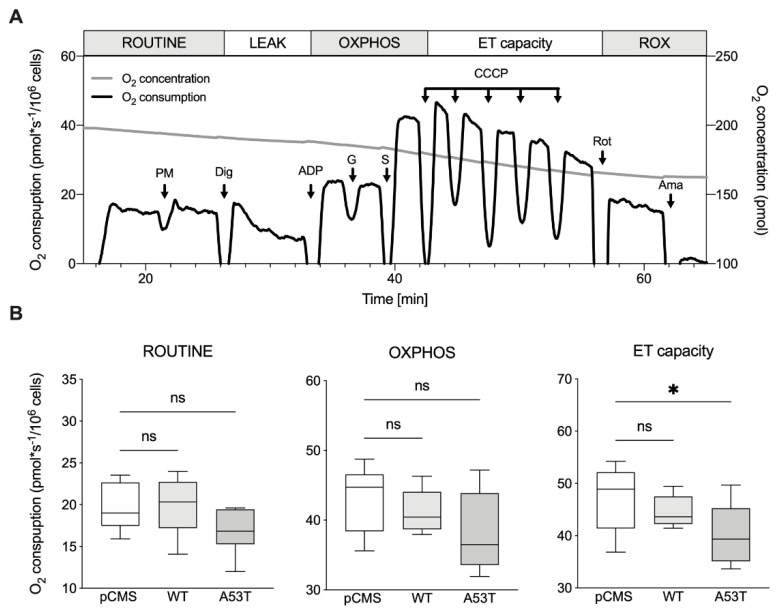
Respiratory profile of transfected SH-SY5Y cells. (**A**) Representative respirometric curve of SH-SY5Y transfected with the empty pCMS showing the oxygen consumption as a function of the time. The SUIT protocol applied and the corresponding respiratory states achieved are also reported. Arrows indicate the addition of substrates: PM, pyruvate and malate; Dig, digitonin; G, glutamate; S, succinate; CCCP, carbonyl cyanide 3-chlorophenylhydrazone; Rot, rotenone; Ama, antimycin. (**B**) Quantitative analysis of the oxygen fluxes obtained for transfected SH-SY5Y in the main respiratory states ROUTINE, OXPHOS and maximal ET capacity. Data are expressed as a median ± SD of *n* = 6 independent experiments and analyzed by one-way ANOVA with * *p* < 0.05; ns, not significant.

The respiratory profile of transfected SH-SY5Y was then analyzed in detail. For this purpose, HRR and a specific SUIT protocol were used, aimed at investigating the oxygen consumption in the main respiratory states. Figure 2A displays a representative respirometric curve of cells transfected with the empty vector showing changes in the oxygen flux following the addition of substrates, inhibitors or uncouplers. According to the protocol, oxygen consumption was measured before plasma membrane permeabilization, i.e., in the presence of endogenous substrates (ROUTINE state), or immediately after, in the presence of saturating ADP concentration and externally added substrates able to stimulate the oxidative phosphorylation (OXPHOS state). The subsequent titration with the uncoupler CCCP, up to the complete dissipation of the proton gradient, allowed us to analyze the maximal capacity of the electron transport system (ET capacity). 

The same protocol was applied to SH-SY5Y cells expressing αSyn WT and A53T mutant and the oxygen flows correspondent to each previously described state were measured (see Table S2 for raw data). As reported in Figure 2B, no significant difference was noticed among our samples in ROUTINE or OXPHOS state. Conversely, the overexpression of αSyn A53T, but not of WT, correlated with a significant reduction of the oxygen flow corresponding to the maximal capacity (−15% vs. pCMS, *p* = 0.04), in agreement with previous observations [21,28]. Cell viability and mitochondrial mass being unvaried (see Figure 1), this result suggests that what we observed is directly related to the overexpression of αSyn A53T.

### 3.3. αSyn A53T Increases Oxidative Phosphorylation Related Flux but Not the ATP-Related One

**Figure 3:**
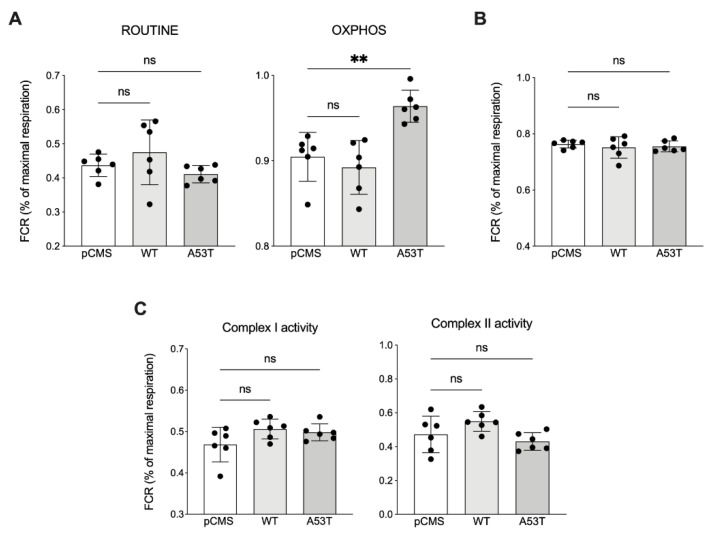
Analysis of the OXPHOS respiration in transfected SH-SY5Y cells. (**A**) Quantification of the specific contribution of ROUTINE and OXPHOS to the maximal ET capacity assayed in transfected SH-SY5Y cells. (**B**) Analysis of the net OXPHOS respiration in transfected cells, corresponding to the ADP phosphorylation flux. (**C**) Quantitative analysis of the specific contribution of complex I and complex II to the maximal ET capacity of transfected SH-SY5Y. Data are expressed as FCRs and as a mean ± SD of *n* = 6 independent experiments. Data were analyzed by one-way ANOVA with ** *p* < 0.01; ns, not significant.

In order to analyze the contribution of each respiratory state or complex to the maximal ET capacity, we analyzed the Flux Control Ratios (FCRs) [25,26]. As reported in Figure 3A, the oxygen rate measured in ROUTINE conditions was comparable among samples. Conversely, after cell permeabilization and upon external stimulation with specific substrates, an unexpected increase of the OXPHOS respiration rate was exclusively observed with αSyn A53T overexpression (+7% vs. pCMS, *p* = 0.015). Being the synthesis of ATP the goal of oxidative phosphorylation, we measured the net OXPHOS respiration, i.e. the oxygen consumption exclusively devoted to ADP phosphorylation. As reported in Figure 3B, no significant differences were observed among samples, suggesting that the previous increase observed in the total OXPHOS respiration does not correspond to a similar increase of ATP-related flow.

Activation of the ET system depends on the activation of complex I, complex II or both. The SUIT protocol described in Figure 2A allowed us to investigate the specific contribution of each complex to the total OXPHOS flux. In particular, the complex I activity was measured before the addition of succinate (a specific activator of complex II), in the presence of pyruvate, malate and glutamate. Complex II activity was instead attained after the addition of succinate and the inhibition of complex I with rotenone. As indicated by the quantitative analysis in Figure 3C, neither the activity of complex I nor that of complex II varied in any condition tested, suggesting that the increased OXPHOS respiration observed in the presence of the αSyn mutant is independent from the functioning of both complexes.

### 3.4. αSyn A53T Increases LEAK Respiration and Diminishes the Excess Capacity of Mitochondria

**Figure 4:**
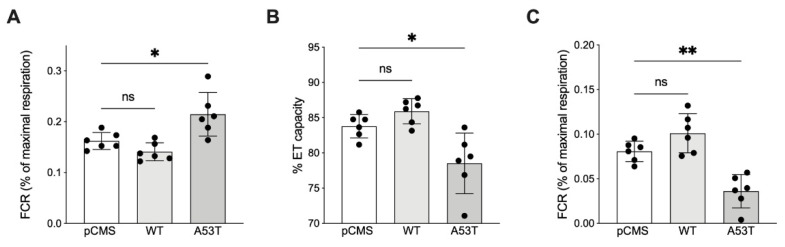
Analysis of the LEAK, coupling efficiency and excess capacity in transfected SH-SY5Y cells. (**A**) Analysis of the specific contribution of LEAK to the maximal ET capacity of transfected SH-SY5Y cells. (**B**) Analysis of the ET coupling efficiency, expressed as a percentage of the total ET capacity. (**C**) Quantitative analysis of the excess capacity of transfected SH-SY5Y. Data are expressed as FCRs or as a percentage of OXPHOS and as a mean ± SD of *n* = 6 independent experiments. Data were analyzed by one-way ANOVA with * *p* < 0.05 and ** *p* < 0.01; ns, not significant.

The coupling between ET activity and ATP production does not match 100% since some protons can partially return to the matrix across the IMM independently from the ATP synthase [29]. The proton leak roughly accounts for 95% of respiration measured in our cells immediately after the mild permeabilization of plasma membranes, the so-called LEAK respiration. According to Figure 2A, in this state, the oxygen consumption is pushed by the activity of the respiratory chain in presence of pyruvate and malate, and simultaneous absence of adenylates [30]. Based on previous results, we queried whether the increased OXPHOS respiration linked to αSyn A53T was due to variation in the dissipative respiratory component. As reported in Figure 4A, a significant increase of LEAK respiration was detected in the presence of the αSyn mutant (+32% vs. pCMS, *p* = 0.017) but not with the WT. Not coincidentally, the percentage of oxygen consumption coupled to phosphorylating events, the ET coupling efficiency, decreased from 84% in control cells to 78% in αSyn A53T expressing cells (*p* = 0.015, Figure 4B). In contrast, cells expressing αSyn WT showed a coupling efficiency of about 86%, thus almost indistinguishable from that of the control. 

Finally, we questioned how much the increase in the dissipative portion of the OXPHOS capacity affected the excess capacity of mitochondria, consisting of the respiratory reserve calculated as the difference between the oxygen consumption in OXPHOS and ET capacity. As reported in Figure 4C, in our experimental conditions, the excess capacity measured in the presence αSyn of A53T mutant was more than halved in comparison to cells expressing αSyn WT or the control (−55% vs. pCMS, *p* = 0.01). Overall, our results indicate that αSyn A53T leads to an increase of dissipative events across the IMM, which, in turn, are responsible for the drastic reduction of mitochondrial respiratory reserve.

## 4. Discussion

The impairment of mitochondrial bioenergetics is widely considered a key event for neurodegeneration. It depends on many factors, including the accumulation of misfolded proteins on the organelle surface, the increased oxidative stress, lipid peroxidation and calcium dysregulation [31,32]. Among misfolded proteins, αSyn is responsible for a broad set of neurodegenerative diseases namely synucleinopathies. The literature indicates that overexpression of the PD-related αSyn A53T drives mitochondrial dysfunction processes [19,20,21], albeit the precise basic mechanism is mostly unknown. 

With this concern, we applied HRR to analyze any specific changes in the mitochondrial respiratory profile of neuroblastoma cells overexpressing αSyn mutant. In our conditions, transient transfection was not sufficient for inducing cell death, as already demonstrated by different groups in SH-SY5Y cells or in other models [27,28], nor mitochondrial mass reduction, as evinced from our experiments. At the same time, the presence of A53T correlated with a reduction of the oxygen consumption relative to the maximal ET capacity, overlapping with literature results attained in cortical primary neurons and N2A cells both expressing αSyn A53T [21,28]. Therefore, transient transfection allowed us to achieve high expression of exogenous αSyn proteins without affecting cell viability or mitochondrial mass, leading us to assume that changes in mitochondrial respiration were exclusively due to a direct effect of αSyn overexpression. 

By applying a SUIT protocol specifically developed for the analysis of the whole respiratory profile of SH-SY5Y [25], we found that while αSyn WT never changed the respiratory parameters, A53T overexpression correlated with a significant and unexpected increase of the OXPHOS respiration, not in terms of absolute values but rather as contributing to the maximal respiration. This result, however, was not linked to increments in the activity of complex I or II, but associated with a proportional increase of the dissipative LEAK component. The phosphorylation of ADP to ATP is strictly coupled to the electron transfer from NADH or FADH2 to oxygen through the respiratory complexes, a mechanism which allows the formation of the proton gradient. However, these two processes are not entirely coupled, due to the return of some protons to the matrix independently from ATP synthase [29]. In fact, OXPHOS capacity (P) is always partitioned into the dissipative LEAK component (L) and ADP-stimulated P-L net OXPHOS capacity, even in physiological conditions. This means that the coupling efficiency is not 100% and part of the proton gradient is dissipated by proton leak processes [29]. 

In general, proton leak depends on the integrity of the IMM and its lipid composition, abundance of ANT and carriers, and activity of uncoupling proteins (UCP) [33]. Under pathological conditions, the proportion of proton leak can increase defining a dyscoupled respiration state. It is thus important to note the difference between the physiological and regulated uncoupling and the pathological dyscoupling, the latter strictly related to mitochondrial dysfunction. Many stress conditions, such as the accumulation of misfolded proteins, increase the partial dissipation of the proton gradient. For example, it has been recently demonstrated that aggregation of SOD1 mutants on the cytosolic surface of mitochondria, as occurred in an amyotrophic lateral sclerosis (ALS) model, increases LEAK respiration [34], while the displacement of SOD1 from the organelle reduces the LEAK flux up to physiological levels [35]. Notably, in the same ALS model, the increment of LEAK affects also the ATP-related flux [35]. In the presence of αSyn A53T we observed a similar increase of the LEAK respiration together with a proportional rise of the OXPHOS one. This allows the cell to counteract the dissipation of the proton gradient and maintain unaltered the ATP-related oxygen flow, as demonstrated by our experiments, thus meeting the energetic needs of the cells. 

It should be considered that cells may use the LEAK respiration as a protective stress response: a mild dyscoupling of the proton gradient, indeed, could attenuate ROS production without impacting on energy production but contributing to prevent the oxidation of proteins and lipids [36]. Our experiments do not clarify whether the increase in the LEAK respiration is a cause or an effect of the presence of αSyn A53T. However, since no variation exists in the flux devoted to the ATP production between A53T and WT or empty vector, we can speculate that the observed increase in LEAK respiration may be a cell response put in place in order to counteract the presence of the mutant protein. To achieve this result, mitochondria draw resources from their excess capacity. Excess capacity is a respiratory reserve on which the mitochondria rely on in case of sudden high energy demands or when stress conditions occur [26]. The magnitude of this reserve can be reduced by an increase in LEAK respiration which, in turn, pushes the OXPHOS capacity toward the limit of the maximal respiration [37]. A contribution to the lessening of the mitochondrial excess capacity is also due to the decrease of maximal respiration itself [37]. These changes in mitochondrial respiration due to the A53T mutant may underlie the cellular susceptibility to additional stress stimuli such as aging and exposure to environmental toxins, as occurred in the case of MPP^+^ exposure [38].

In conclusion, with this work we outlined for the first time the importance of excess capacity for the proper maintenance of mitochondrial function. 

## Data Availability

Not applicable.

## Supplementary Materials

The following supporting information can be downloaded at: https://www.mdpi.com/article/10.3390/life12060894/s1, Table S1: List of primers used in this work for PCR cloning and mutagenesis; Table S2: Raw data of oxygen consumption of transformed cells in different respiratory states.
