# Supplementary material for: α-Synuclein A53T Promotes Mitochondrial Proton Gradient Dissipation and Depletion of the Organelle Respiratory Reserve in a Neuroblastoma Cell Line
Source: Life (Basel). 2022 Jun 15;12(6):894. doi: 10.3390/life12060894 (PMC9225574; doi:10.3390/life12060894)
Supplement: Supplementary file 1 [file life-12-00894-s001.zip › life-1757683-supplementary.pdf]

life

MDPI

Supplementary Materials to:

α-Synuclein A53T Promotes Mitochondrial Proton Gradient Dissipation and Depletion of the Organelle Respiratory Reserve in a Neuroblastoma Cell Line

Pierpaolo Risiglione [1], Salvatore Antonio Maria Cubisino [1], Cristiana Lucia Rita Lipari [1], Vito De Pinto [1,2], Angela Messina [2,3*], and Andrea Magrì [2,3*]

1 Department of Biomedical and Biotechnological Sciences, University of Catania, Via S. Sofia 64, 95125 Catania, Italy; pierpaolo.risiglione@phd.unict.it (P.R.); salvatore.cubisino@phd.unict.it(S.A.M.C.); cristiana.lipari@phd.unict.it (C.L.R.L.); vdpbiofa@unict.it (V.D.P.)
2 we.MitoBiotech S.R.L., Corso Italia 172, 95125 Catania, Italy
3 Department of Biological, Geological and Environmental Sciences, University of Catania, Via S. Sofia 64, 95125 Catania, Italy
* Correspondence: mess@unict.it (Angela Messina); andrea.magri@unict.it (Andrea Magrì)

	Sequence
Cloning megaprimer FW	5′-ttcaattacagctcttaaggctagagtactATGGATGTATTCATGAAAGG-3′
Cloning megaprimer REV	5′-acgagcataatctggaacatcatatggataGGCTTCAGGTTCGTAGTCTT-3′
Mutagenesis A53T FW	5′-GTGGTGCATGGTGTGACAACAGTGGCTGAGAAG-3′
Mutagenesis A53T REV	5′-CTTCTCAGCCACTGTTGTCACACCATGCACCAC-3′

Table S1 List of primers used in this work for PCR cloning and mutagenesis.

	pCMS	α-Syn WT	α-Syn A53T
ROUTINE	19.55 ± 2.81	19.89 ± 3.58	16.90 ± 2.62
LEAK	8.19 ± 1.70	6.60 ± 0.82	7.67 ± 1.40
OXPHOS (Complex I)	21.81 ± 3.80	21.30 ± 2.63	19.04 ± 2.72
OXPHOS (Complex I + II)	43.25 ± 4.70	41.24 ± 3.14	38.31 ± 5.88
ET capacity	47.34 ± 6.18	44.58 ± 2.99	40.30 ± 5.91
ET capacity (complex II)	19.12 ± 2.44	18.05 ± 1.55	20.04 ± 1.90

Table S2 Raw data of oxygen consumption of transformed cells in different respiratory states. Data are expressed as pmol/sec per million cells and reported as a mean ± standard deviation of n=6 independent experiments.
